# Covalent Binding Antibodies Suppress Advanced Glycation: On the Innate Tier of Adaptive Immunity

**Published:** 2009-07

**Authors:** T. Shcheglova, S. P. Makker, A. Tramontano

**Affiliations:** 1 Department of Pediatrics, University of California, Davis - School of Medicine Davis;; 2 Institute of Cytology and Genetics, Siberian Branch of the Russian Academy of Sciences

## Abstract

Non-enzymatic protein glycation is a source of metabolic stress that contributes to cytotoxicity and tissue damage. Hyperglycemia has been linked to elevation of advanced glycation endproducts, which mediate much of the vascular pathology leading to diabetic complications. Enhanced glycation of immunoglobulins and their accelerated vascular clearance is proposed as a natural mechanism to intercept alternative advanced glycation endproducts, thereby mitigating microvascular disease. We reported that antibodies against the glycoprotein KLH have elevated reactivity for glycopeptides from diabetic serum. These reactions are mediated by covalent binding between antibody light chains and carbonyl groups of glycated peptides. Diabetic animals that were immunized to induce reactive antibodies had attenuated diabetic nephropathy, which correlated with reduced levels of circulating and kidney-bound glycation products. Molecular analysis of antibody glycation revealed the preferential modification of light chains bearing germline-encoded lambda V regions. We previously noted that antibody fragments carrying V regions in the germline configuration are selected from a human Fv library by covalent binding to a reactive organophosphorus ester. These Fv fragments were specifically modified at light chain V region residues, which map to the combining site at the interface between light and heavy chains. These findings suggest that covalent binding is an innate property of antibodies, which may be encoded in the genome for specific physiological purposes. This hypothesis is discussed in context with current knowledge of the natural antibodies that recognize altered self molecules and the catalytic autoantibodies found in autoimmune disease.

## INTRODUCTION

The generation of an enormous diversity of antibodies in response to the multitude of possible antigens is a signature of instructive or adaptive immunity. The structural basis for adaptive immunity is expressed in the variability of the antigen binding sites displayed on antibodies and B cell receptors. Thus, antibodies are conventionally associated with the genetic recombination and accumulated mutations in their variable (V) regions that incrementally improve the complementarity between the antibody combining site and groups on the antigen. In contrast to affinity that matures gradually over time through multiple weak interactions, binding through strong forces such as a covalent bond could enable a more rapid and efficient way to capture certain antigens. Is there any case where antibodies use this form of binding and what purpose could such a binding mechanism serve?

Antibodies that bind ligands covalently have been sought in approaches to generate enzyme-like catalytic antibodies (1). Covalent binding is used by enzymes to stabilize reactive intermediates in catalysis of many types of reactions. Reactive immunization was conceived as a strategy to elicit antibodies that bind their ligands through a covalent complex (2). Such antibody complexes might mimic enzyme intermediates to catalyze the transformation of the bound substrate. The premise assumes that this form of binding could be evoked through the conventional affinity maturation process for antibody induction. Implicitly, such antibodies would have experimentally conferred, and therefore artificial, activity. In the prototypical example, immunization against synthetic antigens containing a reactive dicarbonyl group provided antibodies that bind through Schiff base - enamine adducts. The covalently reactive clones were shown to possess remarkable aldolase activity (2). As predicted, the covalent binding function arises from the somatically mutated V region genes, positioning one or more nucleophilic lysine residues in the combining site (3).

## Covalent binding antibodies in glycation and pathology

In an alternative framework one could postulate that covalent binding antibodies might also occur naturally if this activity were advantageous to the host. We proposed that binding through a single strong interaction to an antibody would be an appropriate mechanism for the sequestration and clearance of chemically damaged proteins and cells. Such a function is increasingly recognized in studies of naturally occurring antibodies that have inherent affinity for altered structures on self (4). For example, certain IgM antibodies that compete with macrophage receptors for binding of oxidized LDL particles rely on the recognition of distinct chemical moieties such as the phosphorylcholine headgroup on oxidized phospholipids. These natural autoantibodies (nAbs) are encoded in the germline and typically lack somatic mutations (5). Armed with this "innate-like" reactivity, nAbs are believed to constitute a disposal system for continuous surveillance and elimination of altered self, or "neoantigens" shed from apoptotic cells and damaged tissues (6). The same nAbs also bind to phosphorylcholine groups on bacterial cell wall polysaccharides, thus providing a first line of defense against infections (7). This dual purpose could explain the conservation of this function in the germline repertoire. The molecular basis for the interaction of V regions of nAbs with oxidized phospholipids remains under investigation.

**Fig. 1.**
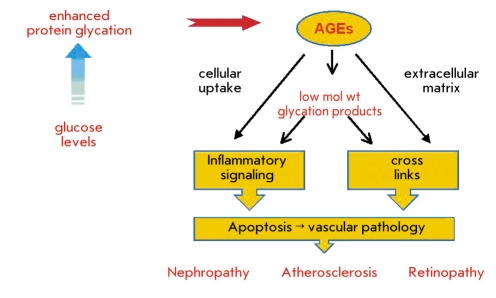
Advanced Glycation Endproducts in Pathology. Protein glycation due to hyperglycemia or normal aging are further modified in the body to advanced glycation endproducts. These AGE may be further broken down to glycated peptides and low molecular weight AGEs. Both high and low molecular weight AGEs could be taken up by vascular tissues by cellular receptors and by cross-linking of the extracellular matrix. These modifications account for cytotoxicity and tissue necrosis and ultimately lead to vascular pathologies as is seen in diabetic complications

**Fig. 2.**
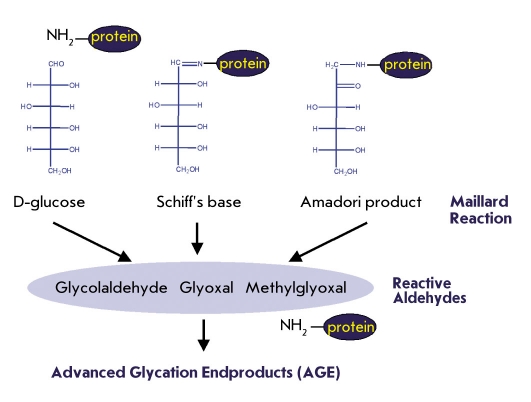
Reactive carbonyls and aldehydes are intermediates in the formation of cytotoxic AGEs. Early glycation products are primarily the result of Amadori reaction of proteins with reducing sugars. Further nonenzymatic fragmentation of these products generate additional reactive aldehydes, which participate in the further protein modifications and cross-linkages know as the Maillard reaction to produce AGEs

Another kind of cytotoxic metabolic waste is generated through glycation or glycoxidation as sugars and carbohydrates are constantly bathing proteins and cells and modifying them through nonspecific reactions of their exposed carbonyl groups. Glycation is a slow and continuous process that occurs in normal aging. However, it is significantly elevated in diabetes due to recurring hyperglycemia or poor glycemic control. The role of this pathway in leading to vascular complication of diabetes is now firmly established (8-10). A bewildering array of advanced glycation endproducts (AGEs) constitutes a class of altered self, which has only been superficially characterized. AGEs initiate pathologies of vascular tissues by two major mechanisms: alteration of the extracellular matrix through protein crosslinks, and the modulation of cellular functions by interacting with specific receptors. Diverse routes of cytotoxicity are suggested by the variety of receptors implicated in AGE uptake, including the receptor of AGE (RAGE), macrophage scavenger receptor, galectin-3 and megalin (11-15). The resulting cellular responses, including plaque formation and tissue restructuring, contribute to the progression of cardiovascular, renal, and microvascular diseases (Figure 1). A number of approaches to therapy of AGE-related pathogenesis are under investigation, including pharmacological inhibition of AGE formation (16) and biopharmaceutical blockade of AGE receptors (17). In principle, a natural homeostatic mechanism to deplete cytotoxic AGEs from circulation could mitigate pathology from ongoing or excessive glycation. Such a mechanism would likely include regulation to boost protection in a stress response. Is there a molecular basis for antibodies to fulfill such a housekeeping function? While AGEs can be highly heterogeneous, the chemical intermediates leading to their formation often bear carbonyl groups derived from the reducing sugars as a distinct chemical signature (18). Initially, carbonyls are introduced through the Amadori reaction and may be retained in various protein adducts generated in the subsequent Maillard reaction. Further degradation of these adducts produces low molecular weight aldehydes, dialdehydes, and glycated peptides, which can react again to modify other proteins (Figure 2). Thus, the concept of "carbonyl stress" has come to denote the chronic pathologies resulting from glycation and oxidation. An obvious mechanism to mitigate cytotoxic AGE formation is to reduce the carbonyl load. A reducing environment within cells allows one level of protection. However, extracellular scavengers might also be expected to protect targets in circulation, on cell surfaces, and in the interstitial space. The efficacy of certain carbonyl-reactive pharmaceutical agents (19) and of monoclonal anti-Amadori albumin antibodies (20, 21) in therapy of diabetic complications provides a further rationale to implicate natural carbonyl scavenging. Covalent binding by antibodies would have distinct advantages in this capacity, which conventional antibody binding could not match. In the hypothetical immune process, a common antibody would recognize diverse modified antigens by strong covalent binding to the carbonyl residue, rather than requiring many antibodies to bind a multitude of conventional epitopes on all the possible glycation products.

A favorable chemical reaction of antibodies with glycation products is suggested by the enhanced glycation of normal immunoglobulins (22-24). Glycated peptides from diabetic animals were shown to react in vitro with normal IgG to modify their L chains (25). Furthermore, glycated L chain is one of three major serum proteins isolated from diabetic subjects (26). Most proteins can undergo glycation to a varying degree, with some proteins more reactive than others. Hence, the modification of immunoglobulin is not surprising in itself, but the selectivity for L chain suggested that highly reactive sites must be available on these polypeptides. The chemical reactivity of L chains could be attributed to their unique sequences found in either the constant or variable domain. The preferential glycation of Fab and Fv (22, 24) and impaired antigen binding of glycated antibodies (23) support the idea that reactive residues are located in the variable sequences. Accordingly, reactivity might also be modulated by somatic diversification of V region sequences of the L chains.

## Induction of covalent binding antibodies

**Fig. 3.**
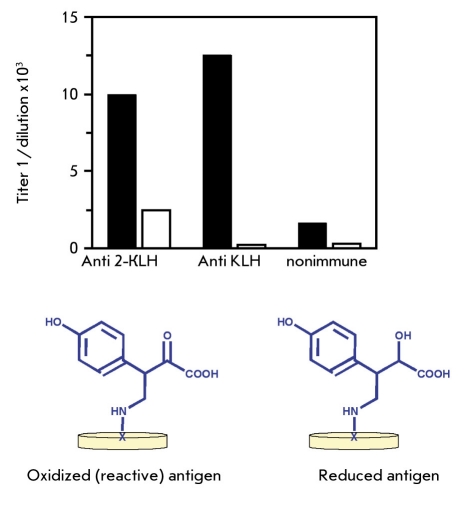
Covalent-binding ELISA. Antibodies induced against a pyruvate-KLH conjugate (anti-2 KLH) or against unmodified KLH reacted specifically by ELISA with the pyruvate (2) coupled to BSA (filled bars). Binding to chemically reduced glycolate-BSA (open bars) could be observed with anti-2 KLH antibodies but not with anti-KLH antibodies. Normal, non-immune IgG bound poorly to either molecule. We hypothesized that this binding is mediated by a reaction resulting in covalent binding of the pyruvate carbonyl group with anti-KLH antibodies

**Fig. 4.**
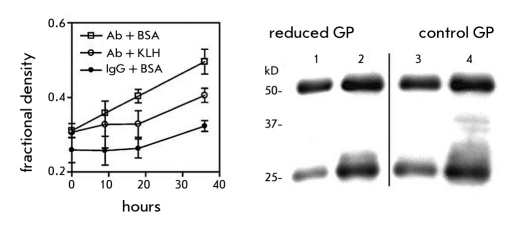
Reaction of anti-KLH antibodies with glycated peptides from diabetic serum. (Right panel) The density of the bands for products at 30- 34 kDa detected by SDS-PAGE and immunoblot analysis is plotted as a fraction of the total L chain density. Formation of L chains of higher mass was observed in the reaction of antibodies from normal and KLH-immunized rats. The rate of the latter was inhibited in the presence of KLH. (Left panel) The reaction of L chains with glycopeptides from diabetic rat serum at 0 h (lanes 1 and 3) and 24 h (lanes 2 and 4). Glycopeptides that were reduced by sodium borohydride failed to react in this time (lanes 1 and 2), while the untreated glycopeptides showed enhanced formation of L chain adducts at 30 - 34 kDa (lanes 3 and 4)

In order to test this hypothesis we sought to demonstrate that covalent binding antibodies could be elevated in an immune response and that these could attenuate a glycation stress by reducing the carbonyl load. Reactive immunization suggested a feasible approach. Previously, we showed that antibodies induced to a pyruvate-containing hapten-KLH conjugate could bind to antigen by recognizing only the carbonyl group in the hapten (27). However, to our surprise the anti-KLH antibodies accounted in large part for this reactivity. Antibodies to KLH bound to the reactive pyruvate in the same way as established by differential binding to the pyruvate/glycolate hapten pair (Figure 3). Covalent Schiff base formation between antibody and the pyruvate carbonyl was the most plausible explanation for this focused binding. To test whether these antibodies could also neutralize carbonyl groups on glycation products, we compared anti-KLH antibody and normal IgG in the reaction with glycated peptides from sera of diabetic rats. In fact, this assay showed that L chains of anti-KLH antibody were more reactive than L chains of normal IgG (28). The chemically reduced glycated peptides failed to form covalent adducts, indicating that carbonyl groups were necessary for the reaction (Figure 4). Proteomic analysis of the modified L chains by tandem mass spectrometry showed V region peptides derived from only two lambda L chains, even though kappa L chains comprise more than 90% of rat IgG. Remarkably, these lambda L chain peptides revealed sequences identical to the germline-encoded VL (28). However, these results also suggested that L chain reactivity was enhanced by immunization. Although mass analysis detected only peptides of unmutated germline VL, modified peptides with mutated residues might not have been identified by proteomic analysis. Alternatively, L chains with innate reactivity might be recruited in combination with somatically diversified VH domains, which provide specificity for KLH and cross-reactivity for glycated peptides.

## Reactive antibodies ameliorate glycation-associated pathology

The streptozotocin-induced diabetic rat, serving as a suitable model for diabetic nephropathy, was used to demonstrate the potential of the reactive immune response to mitigate AGE formation in vivo. Compared to diabetic animals immunized with adjuvant alone, KLH-immunized diabetic rats had significantly diminished AGE levels, which correlated with attenuated nephropathy (28). Improved renal function, as determined by glomerular morphology and proteinuria levels, was accompanied by reduced AGE staining in the renal extracellular matrix. We hypothesized that the therapeutic benefit derived from improved capture of glycation products in circulation by the reactive antibodies. This mechanism presumes that AGEs formed from antibody molecules are less cytotoxic than the alternative protein AGEs. Formation of glycated IgG in diabetic subjects and, in particular, the identification of glycated L chain in the diabetic serum (26) indicated that these molecules might play a role in AGE pathogenesis. Although modified L chains accumulated in the reaction with glycated peptides in vitro, these products were not significantly elevated in the serum of diabetic animals. Glycated IgG is cleared from circulation and taken up by kidney more efficiently than unmodified IgG (29). Furthermore, the filtration properties of low mass glycated proteins favor their selective excretion (30). Thus, native and modified L chains are found in the urine of both healthy and diabetic subjects (31, 32). Taken together, these observations are consistent with the hypothesis of enhanced clearance of selectively glycated antibodies.

These studies provide evidence that a natural covalent reactivity of antibodies is augmented by adaptive immunity. However, the predominance of unmutated V region sequences in the reaction products suggested that reactivity is inherent to the germline encoded VL (28). How could reactivity be enhanced by affinity maturation, yet conserved in an unmutated germline configuration? Glycosylated residues of KLH are believed to account for the cross-reactivity of anti- KLH antibodies for carbohydrate epitopes of microbial antigens (33). Similarly, anti-KLH antibodies may also cross-react against glycosylated epitopes of tumor antigens, as suggested by the therapeutic benefits of KLH in bladder carcinoma (34). Thus, a carbohydrate-specific response could account for the specificity of KLH antibodies for glycated peptides. This specificity could be imprinted in the H chain, which when paired with a nonspecific, reactive L chain would enhance reactivity of the latter against the bound substrate. The structures of reactive Ig V regions and the adaptive mechanisms guiding their reactivity remain subjects for further investigation.

## Covalent binding antibodies from reactive selection and reactive immunization

**Fig. 5.**
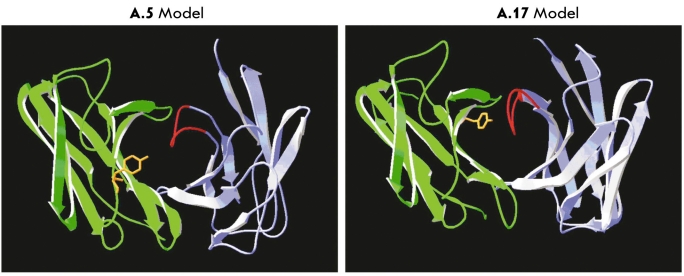
Molecular models of the variable regions of covalent binding antibody Fv fragments. The yellow side wire model indicates the side chain Tyr residues participating in nucleophilic attack on the organophosphorus reagent. The Tyr at VL32 in A.5 is part of CDR1 that is oriented near the surface of the Fv domain, whereas Tyr at VL37 in A.17 is in FR1 at the interface between VL and VH. This model implicates a conserved active site in a deep cleft on A.17

In an alternative approach, we used synthetic reactive substrates as antigens to probe for antibodies capable of covalent binding. This idea followed the original concept that antibodies selected for nucleophilic reactivity could also express enzymatic activity through covalent catalysis. Irreversible covalent binding to a small organophosphorus (OP) ester was used to chemically select single-chain VH-VL fragments (scFv) from a phage display library (35). All of the selected chemically reactive scFv molecules were modified on the VL polypeptide and could be described by two canonical sequences. The more reactive clone A.17 used the DPL-5 germline VL product, which was phosphonylated at Tyr37 within the framework region FR-L2. By contrast, six other reactive clones used the DPL-3 germline VL, which reacted at Tyr32 in CDR-L1. These nucleophilic Tyr residues are conserved in the VL germline and three-dimensional models of the scFv suggest that either residue can be oriented toward the combining site. However, the models also indicate that the Tyr37 is buried at the interface between VL and VH domains in A.17, where it could be sterically inaccessible to ligand contact without large conformational motions (Figure 5). Since the library was constructed by shuffling of germline VL and VH gene segments, we did not expect natural pairs in the scFv (36). Nevertheless, the VH chains of reactive clones were represented primarily by highly homologous sequences belonging to the VH4 family. These results strongly suggested that the VH chain plays an important part in enhancing the chemical reaction at residues on the VL region. The scFv could also bind other structurally unrelated OP compounds indicating a lack of fine specificity for ligand structure (35). The A.17 scFv was also shown to have modest hydrolytic activity for peptide amides and simple carboxylic ester substrates. These Ig V regions could thus serve as primitive covalent catalysts. Most intriguing is the notion that the reactivity emerges from certain germline-encoded VL-VH pairs. Additional studies of these monoclonal Fv fragments will be of interest for understanding the origins and biochemical functions of chemically reactive Ig molecules.

Chemical selections in vitro might represent the first step in adaptive immunity for acquisition of reactivity. However, the contribution of irreversible covalent binding to clonal selection in vivo remains speculative. Using a reactive OP esterprotein conjugate as immunogen, we examined the potential for the immune response to elicit reactive antibodies that are modified by the antigen. Antisera against the OP conjugate included reactive antibodies, which could be detected by their covalent modification with biotin-tagged OP reagent (27). Remarkably, these polyclonal antibodies were also modified predominantly on their L chains. Thus, it appeared that the reactivity was enhanced by affinity maturation. However, it is not clear whether the covalent binding contributed to clonal selection or whether it is merely incidental to the affinity matured antibodies. Considered as an enzyme system, the rate of modification (antigen capture) should increase as the noncovalent binding, expressed by the Kd of the Michaelis-like Ab*OP complex, increases. While affinity maturation does not require it, an increasing chemical rate (ks) of irreversible covalent modification (Ab-X) might provide kinetic selection for clonal expansion of B cells (Scheme I). Ultimately, the question is whether covalent capture of antigen is adopted in a natural function for immunity or for host survival.

**Scheme I.**
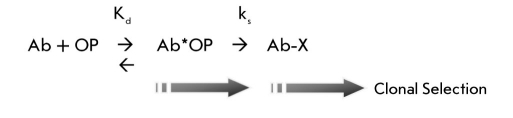


## Carbonyl chemistry of oxidation-derived epitopes for natural antibodies

IgM nAbs are predominantly produced by a population of long-lived, self-replenishing B-cells, including the B-1 and B-1a subsets. It is believed that this B cell repertoire is conserved in evolution for its contribution to host defense (5). The germline-encoded nAbs are best known for their capacity to bind conserved determinants on pathogens, referred to as pathogen-associated molecular patterns (PAMPs). More recently it was shown that nAbs also bind to altered epitopes on apoptotic cell and self proteins (37). These findings support the concept of a homeostatic function of nAbs for clearance of debris from cell death and protein decay (6, 38). Chemical structures generated by oxidation and glycoxidation of membrane phospholipids are thus the natural targets for nAb binding, presenting a case where carbonyl chemistry intersects with immune recognition. The phosphorylcholine (PC) headgroup of oxidized phospholipids and PC residues on the bacterial cell wall polysaccharides provide a common molecular determinant for the immune and homeostatic functions of nAbs (7). As a molecular receptor of PAMPs, the nAb V region can be regarded as akin to the evolutionarily conserved Toll-like receptors (TLR) of innate immunity (39). This emerging paradigm has far-reaching implications for the linkage between innate and adaptive immunity.

Oxidized phospholipids, which accumulate in atherosclerotic lesions, are targets of both innate and adaptive immunity (40). The role of reactive oxygen chemistry in defining epitopes for nAbs or for somatically diversified antibody is a subject of continuing investigation. Oxidation in the syn-2 unsaturated fatty acid chain of the phospholipids introduces reactive species, including aldehyde functional groups, into the lipid moieties. One hypothesis suggests that the carbonyl group serves as the reactive anchor for modification of self by the phospholipid moieties. Small aldehydes that are byproducts of lipid peroxidation, such as 4-hydroxynonenal and 4-hydroxyhexenal, are also implicated in protein damage and cytotoxicity and may play a role in autoimmune responses (41). Moreover, malondialdehyde, glycolaldehyde, and other reactive aldehydes generated from early glycation also contribute to LDL modification and to the production of autoantibodies in pathologic conditions (42). The reaction of small aldehydes with residues on the protein surface, including lysine and histidine side chains, might define the neoepitopes of autoantibodies emanating from the nAb population. For example, it has been reported that antibodies induced to histidine adducts of lipid oxidation-derived aldehydes also bind to DNA (43). Structural mimicry between these adducts and 2-deoxyribonucleosides was suggested to explain this DNA binding. These antibodies have high sequence homology to natural anti-DNA autoantibodies and are related to the polyreactive nAbs. Thus, the role of carbonyl residues in promoting immune responses to modified self may have several dimensions. In an expanded context, proteins modified by two or three carbon aldehydes or other reactive small molecules often define ligands for innate immune receptors such as macrophage scavenger receptor and RAGE (44). Although covalent binding of aldehyde-modified proteins to scavenger receptors through Schiff base or carbinolamine adducts was contemplated the early investigations, such interactions between modified self and innate immune receptors, and antibody paratopes in particular, remain to be demonstrated.

## Chemical reactivity, catalysis and the basis for polyreactive antibodies

Polyreactivity of nAbs is defined as the promiscuous avidity of multivalent IgM molecules to disparate molecules, including intracellular proteins and nucleic acids (45). This binding capacity, which can also be manifested as autoreactivity to self, remains poorly understood in structural terms. Enhanced avidity by the recognition of repetitive structures on self molecules or membranes provides a plausible molecular mechanism for polyreactivity. Within this conceptual framework, the repetitive structures suggest another form of molecular pattern identifying the damaged cells and tissues. Thus, the high density of PC groups on oxidized phospholipids and on cell wall polysaccharides offers a common feature for nAb binding. Similarly, phosphate linkages or nucleotide sequences on DNA strands could represent the molecular pattern for DNA antibodies to recognize surface features of apoptotic cells (46, 47).

Reports showing that DNA autoantibodies found in lupus or other autoimmune diseases have phosphodiesterase activity suggests a further case where molecular recognition in the Ig V domains is manifested as chemical reactivity (48). These antibodies utilize their binding site residues to promote the cleavage of DNA ligands. A remarkable feature of the DNA-hydrolytic autoantibodies is their dependence on metal ions, reminiscent of the natural DNAse enzymes (49, 50). However, these enzyme-like antibodies show little DNA sequence specificity, hydrolyzing single-stranded and doublestranded substrates promiscuously (49, 51). Structural studies proposed that a catalytic site for DNA-hydrolysis could be encoded in a VL domain having a near-germline configuration (51). In a more recent example, it was suggested that mutations in VH or VL that enhance DNA binding could also contribute to hydrolytic activities (49). It is well established that anti-DNA IgG autoantibodies are significantly diversified by antigen-driven adaptive immunity (52, 53). For example, positively charged lysine and arginine residues in the combining sites improve binding for anionic DNA. Yet, these residues could also arise from biased VH or VL germline gene usage (54, 55). X-ray crystallographic structure studies reveal that DNA-binding autoantibodies interact with DNA through the conventional combining site comprised of CDR loops (56, 57). Mutations in both VL and VH contribute residues for direct antigen contact or for conformational changes that indirectly improve complementarity. Nevertheless, the ontogeny of DNA autoantibodies could also reflect the inherent DNA binding activity of nAbs. DNA binding can evolve directly from the PC recognition function by a single mutation in the murine monoclonal nAb T15 (58). Earlier reports also showed that DNA autoantibodies have restricted VH family usage (59) and can be encoded entirely by germline V genes without any somatic change (60-62). Reactivity of the germline-encoded V region has also been proposed to account for proteolytic activity of natural autoantibodies (63). Collectively, these findings strengthen the hypothesis that catalytic activities of Ig V regions exist as an innate immune function. The focus on polypeptide or oligonucleotide substrates for catalytic autoantibodies obscures the distinction between innate and adaptive immunity, as these substrates can engage in extended interactions with the conventional antigen combining sites. Specificity for extended features of autoantigens implies the work of adaptive immunity. However, broad reactivity without regard for the substrate structures flanking the cleavage site suggests promiscuity of a primitive active site. In depth biochemical and structural investigation of the inherent reactive or catalytic sites of natural antibodies will further illuminate the biological significance of the chemical function.

## Conclusions

Covalent-binding antibodies obtained by immunization or reactive selection and catalytic antibodies found in autoimmune pathologies appear to have features in common with nAbs. These features include VH and VL domains in germline or near-germline configurations and the recognition of molecules presenting uncommon chemical functionality, such as reactive carbonyls and phosphorus esters. Such features may be fundamentally related in that the chemical or enzymatic function is presumably highly evolved and therefore conserved in the genome. Whereas nAbs are IgMs that rely on weak multivalent binding to molecular patterns on antigens for high avidity, chemically reactive or catalytic antibodies are also expressed as IgG that can bind antigens with high affinity. However, the latter may also react with diverse substrates through weak, noncovalent pre-reaction complexes. According to the transition state theory of catalysis the strongest binding is expressed between an enzyme and the transition state. In the covalent complex, binding is dominated by the chemical bond between a residue of the active site and a group on the ligand. The focus of the weak binding by nAbs on small, minimally altered epitopes is consistent with the recognition of substrate functionality for reactivity. Thus, chemical reactivity could provide a mechanism to translate weak non-covalent binding to strong binding or to catalytic activity. The physiological purpose of chemically reactive and catalytic antibodies must also be addressed, even as the role of nAbs in protective immunity and homeostasis is only beginning to emerge. Participation of nAbs as response elements in oxidative stress and apoptotic cell clearance suggests a housekeeping function that predates the evolution of adaptive immunity. Similar considerations may apply to the rationale for covalent-binding antibodies acting as buffers against glycation stress. The preservation of covalent reactivity or catalytic functions in adaptive responses and in autoimmunity could be attributed to biological design or to defects in immune regulation. Inducibility through adaptive immunity might offer an appropriate mechanism to invoke beneficial responses to oxidative or glycoxidative stress. On the other hand, the role or contribution of natural catalytic autoantibodies in autoimmune pathology remains obscure, and their existence could simply reflect a failure in the V gene repertoire shift on induction of IgG autoantibodies (64, 65). Designed catalytic antibodies can also be educed from the adaptive immune response by affinity maturation that is appropriate for substrate reactivity or transition state binding. To what extent do designer catalytic antibodies co-opt the functions of natural autoantibody catalysts? An important clue was provided in an earlier report indicating a high frequency of antibodies with hydrolytic activity among hybridomas sampling an autoimmune repertoire (66). Continuing studies of antibody chemical reactivity induced by immunization or discovered in the germline repertoire should provide further insights into its role in immunity or pathology and could enable technological applications of this unconventional antibody function in the future.

## Acknowledgements

This work was supported by the American Diabetes Association grant 1-05-RA-136, NIH grant CA90564, and the UC Davis, Medical School - Children's Miracle Network.
